# Is nigrostriatal dopaminergic deficit necessary for Holmes tremor to develop? The DaTSCAN and IBZM SPECT study

**DOI:** 10.1007/s00702-017-1780-1

**Published:** 2017-08-23

**Authors:** Agata Gajos, Sławomir Budrewicz, Magdalena Koszewicz, Małgorzata Bieńkiewicz, Janusz Dąbrowski, Jacek Kuśmierek, Jarosław Sławek, Andrzej Bogucki

**Affiliations:** 1 Department of Extrapyramidal Diseases, Central University Hospital, Medical University of Łódź, Pomorska 251 Str, 92-213 Łódź, Poland; 2 Department of Neurology, Wroclaw Medical University, Wroclaw, Poland; 3 Department of Quality Control and Radiological Protection, Medical University of Łódź, Łódź, Poland; 4 Department of Nuclear Medicine, Medical University of Łódź, Łódź, Poland; 5 Department of Neurological and Psychiatric Nursing, Medical University of Gdańsk, Gdańsk, Poland

**Keywords:** Holmes tremor, SPECT, DaTSCAN, IBZM, Dopaminergic nigrostriatal system

## Abstract

Holmes’s tremor (HT) is assumed to be the result of coexistence of nigrostriatal dopaminergic system impairment and the lesion of cerebello-thalamic pathways. It was suggested that dopaminergic deficiency is responsible for rest tremor, and lack of compensatory cerebellar function leads to spill of tremor into voluntary movements. Cases of HT with and without abnormalities of the presynaptic part of dopaminergic nigrostriatal were published and these findings raised the question of possibility of the postsynaptic lesion. Three patients with HT diagnosed according to criteria of Consensus Statement on Tremor were studied. In all of them SPECT imaging with ligands of presynaptic (I 123-FP CIT—DaTSCAN) and postsynaptic (I 123-iodobenzamide—IBZM) nigrostriatal dopaminergic neurons was performed. Indices of uptake in caudate and putamen normalized to nonspecific uptake in occipital cortex and indices of asymmetry for each whole striatum as well as for putamen and caudate separately were calculated. SPECT studies did not reveal asymmetry of DaTSCAN and IBZM binding in striatum in all studied subjects. The current clinical diagnostic criteria of HT are presumably insufficiently specific and when using them we identify patients both with and without the involvement of dopaminergic system. These two groups may represent tremor disorders of similar phenomenology but of different pathomechanism.

## Introduction

Holmes tremor (HT) is a disorder characterized by the presence of rest, intention and postural tremor of an upper extremity, usually of low frequency (less than 4.5 Hz) and irregular (Deuschl et al. 1998; Deuschl and Bergman 2002; Vidailhet et al. 1998). It was suggested that coexistence of nigrostriatal system and cerebello-thalamalo-cortical or dentate-rubro-olivary connections lesions are necessary for HT to develop (Deuschl et al. 1999). In several cases of HT the unilaterally impaired activity of presynaptic part of nigrostriatal dopaminergic system has been demonstrated by PET and SPECT studies. However, we published a series of 6 HT patients without abnormality showed by SPECT with the use of ^123^I-FP CIT (DaTSCAN) (Gajos et al. 2010). Normal SPECT images obtained with dopaminergic presynaptic tracers raise the question of possibility of the postsynaptic lesion. The aim of this study was to evaluate the pre- and postsynaptic part of nigrostriatal dopaminergic system by means of SPECT-DaTSCAN and SPECT with ^123^I-iodobenzamide (IBZM) imaging, respectively.

## Patients and methods

### Ethics statement

The protocol of the study was approved by the Bioethical Committee at the Medical University of Lodz (permit no.: RNN/110/10/KB). Written informed consent has been obtained from all studied individuals.

### Subjects

Three patients [a part of material presented by our group (Gajos et al. 2010)] with HT diagnosed according to criteria of Consensus Statement on Tremor (Deuschl et al. 1998) were studied.

*Case 1* A 36-year-old male suffered from severe head injury in car accident at the age of 10. The brainstem contusion and subarachnoid hemorrhage was diagnosed (the latter confirmed by CSF examination), however, CT could not be done at that time. The patient was unconscious for several days and later the transient slight weakness of the right arm was observed. One month after the head trauma the paresis resolved and subsequently the patient developed progressive tremor of the right upper limb. CT performed 1 year later did not show any abnormalities. Twenty five years after his first symptoms the patient presented severely disabling rest tremor involving the whole right upper extremity, proximally more prominent. Irregular, low frequency tremor was also present during posture and intentional movements. Emotional enhancement was observed. The patient was unable to perform any task with his right hand and often he had to use his left hand to suppress the affected limb. Tremor did not respond to single dose of levodopa (200 mg). The patient was treated without remarkable effect with piracetam, biperiden, trihexyphenidyl, clonazepam, diazepam, valproate and tiapride. He refused the proposal of DBS. MRI and DaTSCAN-SPECT performed at age of 35 were unremarkable. IBZM-SPECT was done 13 months later.

*Case 2* A previously healthy female patient without any vascular risk factors at the age of 27 suffered from ischemic stroke with hemiparesis involving the left extremities. Hemiparesis improved gradually over the next weeks leaving only residual weakness. Six months later tremor of her left arm was noticed. MRI scan performed at this time showed one ischemic lesion involving the parasagital part of the right temporal and occipital lobe and the second small one in the right thalamus. CT-angiography revealed additional pericallosal artery but no other abnormalities. One year after the stroke neurological examination showed slight (MRC 4/5) weakness and exaggerated deep tendon reflexes on the left side. Discrete ataxia was more pronounced in the lower left limb than in the upper one. Moreover, irregular, low frequency (3–4 Hz) tremor of the left upper extremity, distally more prominent, was observed. It was present at rest and it increased with posture and during intentional movements. Additionally, subtle dystonic (flexion–extension) movements of the left hand fingers were present. Dopaminergic drugs were not administered. DaTSCAN-SPECT and IBZM-SPECT were done, respectively, 24 and 35 months after stroke.

*Case 3* A 41-year-old woman suddenly developed left side hemiparesis and hemianesthesia. CT scan showed ischemic lesion within the dorsal part of the right thalamus. During the next year weakness resolved gradually and tremor in the left upper limb occurred.

Thirteen years later, on neurological examination, an irregular, coarse tremor of the left upper extremity, proximally more pronounced, was found. It was present at rest and with posture and it was enhanced by voluntary movements. Mild left hand dystonia could also be observed. Muscle power was 5/5 (MRC) in the upper and 4/5 in the lower left extremity, muscle tone was normal. Deep tendon reflexes were symmetrically normal, and the plantar responses were bilaterally flexor. Mild cerebellar signs were noted in the left extremities. Superficial sensation of light touch and pain were diminished on the left side. MRI scans revealed an infarct of the right thalamus and scattered small lacunar lesions involving subcortical and periventricular white matter. She did not improve on levodopa (400 mg daily). SPECT studies were performed 12 years after stroke.

### SPECT studies

SPECT/CT acquisitions were performed with double-head hybrid gamma-camera Infinia Hawkeye GE 4 h after i.v. administration of 5 mCi 123I–DaTSCAN and 2 h after the administration of 5 mCi 123I–IBZM. Prior to radiotracer injection, patients received oral potassium iodine (Lugol’s solution) to block thyroid uptake of free radioactive iodide.

Data were acquired with use of low energy high resolution (LEHR) collimators in dual energy window: 159 keV ± 10% (scatter: 130 keV ± 10%), in 128 × 128 matrix. In a step-and-shoot method 120 projections for 45 s each were registered with use of zoom equal 1.5.

Images reoriented to orbitomeatal plane were reconstructed with OSEM method (2 iter., 10 sub., postfilter: Butterworth 0.50/10) with scatter correction and attenuation correction based on CT attenuation maps.

Intensity and symmetry of radiotracer uptake was assessed visually and semiquantitatively by nuclear medicine specialist on transversal slices of striatum. Indices of uptake in caudate and putamen normalized to nonspecific uptake in occipital cortex were calculated with the use of semi-automatic procedure with fixed templates of putamen and caudate as regions of interests (ROIs)—on 3 consecutive transversal slices with most intense striatal uptake.

We used the reference values for SPECT-DaTSCAN developed by Nicastro et al. (2016). They applied a new approach designed to minimize intercenter variability and suggested that their reference limits, established using the percentile approach, may be used in any individual nuclear medicine center.

Indices of asymmetry (IA) for the whole striatum as well as for putamen and caudate separately were calculated according to formula: ((*R* − *L*)/(*R* + *L*)) × 2 × 100 (where *R* is the side with the higher uptake and *L* is the side with the lower uptake, to get a positive value).

Age-corrected lower reference limits for the radiotracer uptake were calculated as follows: striatum 3.78883 − (0.02156 × age); caudate 3.87465 − (0.02141 × age), putamen 3.65557 −(0.02168 × age).

## Results

The semiquantitative evaluation of DaTSCAN and IBZM binding in striatum as a whole and in putamen and caudate separately did not reveal asymmetry in all studied subjects (Table 1). According to Nicastro et al. (2016) IA can be considered normal for the whole striatum when lower than 14.37% (below the 95th percentile). The value of IA for DaTSCAN studies in our subjects was in the range of 2.0–9.0%. Also for the striatum and caudate assessed separately this upper limit was not exceeded in our patients.

**Table 1:** DaTSCAN and IBZM SPECT imaging

Patient (age at scan—years)	Structure	Indices of radiotracer uptake		IA (%)
		Contralateral to HT	Ipsilateral to HM	
DaTSCAN				
1 (35)	Striatum (*N* > 3.03)	5.82	5.98	2.7
	Putamen (*N* > 2.92)	6.03	5.94	1.6
	Caudate (*N* > 3.13)	5.45	6.05	6.3
2 (29)	Striatum (*N* > 3.16)	9.56	9.38	2.0
	Putamen (*N* > 3.03)	9.68	9.13	5.8
	Caudate (*N* > 3.25)	9.36	9.83	4.8
3 (54)	Striatum (*N* > 2.62)	7.23	6.61	9.0
	Putamen (*N* > 2.48)	7.21	6.38	12.2
	Caudate (*N* > 2.72)	7.26	6.73	7.6
IBZM				
1 (36)	Striatum	2.34	2.38	1.6
	Putamen	2.33	2.39	2.6
	Caudate	2.35	2.37	1.0
2 (30)	Striatum	2.96	2.83	4.4
	Putamen	3.02	2.81	7.2
	Caudate	2.84	2.87	1.0
3 (54)	Striatum	2.14	2.16	0.9
	Putamen	2.17	2.13	1.8
	Caudate	2.09	2.21	5.6

Indices of radiotracer uptake in striatum (normalized to nonspecific uptake in occipital cortex) and Index of Asymmetry (IA). DaTSCAN lower limit values, given in parentheses, calculated for striatum, putamen and caudate according to according to Nicastro et al. 2016

We wanted also to exclude the presence of a symmetrical presynaptic nigro-striatal dopaminergic deficit that could be co-responsible for HT development in the event of unilateral damage of another structure. In all three our subjects the age-corrected DAT tracer binding in the whole striatum, putamen and caudate was evidently above the proposed lower limits (Table 1).

## Discussion

In HT rest, postural and goal-directed tremors coexist (Deuschl et al. 1998; Deuschl and Bergman 2002; Vidailhet et al. 1998). It was proposed that concurrent functional impairment of both, nigrostriatal dopaminergic and cerebello-thalamic systems is necessary for HT to occur. Deuschl et al. (1999) suggested that dopaminergic deficiency is responsible for rest tremor, and lack of compensatory cerebellar function leads to spill of tremor into voluntary movements. This hypothesis is strongly supported by observations of patients with Parkinson’s disease who presented HT as a result of cerebellar infarct (Kim et al. 2009) and of patients with preexisting cerebellar lesion who developed HT as a consequence of Parkinson’s disease (Deuschl et al. 1999).

Results of functional neuroimaging studies and responsiveness to dopaminergic agents could be also considered as markers of nigrostriatal deficiency in HT. However, these data are inconclusive.

In Remy et al. (1995) published results of PET evaluation of dopaminergic nigrostriatal system in six patients with “peduncular rubral tremor” of different origin. In all subjects significant ^18^F-dopa uptake reduction was found in striatum ipsilaterally to the brain lesion. It was even more (3–38% of the control values) severe than in Parkinson’s disease. There was no asymmetry in binding of ^76^Br-lisuride, a D_2_ receptor tracer. These results reflected impaired function of presynaptic part of dopaminergic nigrostriatal system and intact the postsynaptic one. All patients responded to dopaminergic therapy: levodopa alone (three cases) or given together with dopamine agonist (three cases). The response was “complete or near complete” in two subjects and “important but incomplete” in another two. However, the analysis of clinical data raises some suspicions, that in some of patients Parkinson’d disease or other form of parkinsonism could coexist. In four of six studied subjects tremor was most severe at rest and in one of them no other forms of tremor were present while in most of published cases of HT it was exaggerated by goal-directed movements. Moreover, in three cases bradykinesia was noted ipsilaterally to tremor and in one of these subjects bilateral rigidity was present.

In the following years several case reports of HT patients studied with SPECT-DaTSCAN were published. In two patients (Paviour et al. 2006; Strecker et al. 2007) reduction of radiotracer binding, more pronounced in the putamen than in the caudate nucleus, was observed. In two other patients SPECT-DaTSCAN did not reveal any tracer uptake suggesting complete unilateral presynaptic dopaminergic denervation (Guedj et al. 2007; Seidel et al. 2009). Our group presented 6 patients, who fulfilled diagnostic criteria for Holmes tremor, without any evidence of presynaptic nigrostriatal dopaminergic dysfunction as shown by SPECT-DaTSCAN (Gajos et al. 2010). On the other hand, in a recently reported case PET with 18F-PR04.MZ, a new high-affinity radiotracer for dopamine transporters, demonstrated significantly reduced binding of ligand within striatum and intact dopaminergic activity of the substantia nigra; reduction of tremor severity after levodopa was noted in this subject (Juri et al. 2015).

Apart from the material presented by Remy et al. (1995) there was, to our knowledge, only one report of case in which both, pre- and postsynaptic parts of nigrostriatal system were evaluated and results of DaTSCAN-SPECT as well as IBZM-SPECT were normal (Hertel et al. 2006). It is consistent with results obtained in our three cases. It cannot be excluded that cases with confirmed dysfunction of the dopaminergic system are overrepresented in the published papers as supporting the HT dopaminergic hypothesis.

Various responses to dopaminergic therapy in patients with HT were observed. According to one report (Raina et al. 2016) 13 out of 24 patients treated with levodopa improved and in seven tremor disappeared almost completely. In some subjects even single dose of levodopa produced dramatic response (Raina et al. 2007; Vélez et al. 2002) and with daily doses between 750 and 1000 long term improvement was achieved (Kim et al. 2009; Raina et al. 2007; Vélez et al. 2002; Pezzini et al. 2002). A good response to levodopa (100 mg tid) persisted for 4 years was reported in a case of HT related to midbrain dysplasia (Boelmans et al. 2012).

On the other hand in several patients levodopa given in dose 300-1500 mg was completely ineffective (Akkus and Diramali 2006; Baysal et al. 2009; Kim et al. 2002, Rieder et al. 2003; Teive et al. 2002; Walker et al. 2007; Zhong et al. 2007). Good response to 2.8 mg of pramipexole was obtained in patient unresponsive to levodopa (Strecker et al. 2007). In one patient 750 mg of levodopa failed to be effective, but 2 mg of cabergoline significantly alleviated all components of tremor (Akkus and Diramali 2006). In some patients dopaminergic therapy was more effective in reducing resting tremor than the kinetic one (Kim et al. 2009; Strecker et al. 2007; Raina et al. 2016; Pezzini et al. 2002) but in others levodopa improved resting, postural and kinetic tremor (Raina et al. 2007). Only limited data concerning the effectiveness of dopaminergic therapy in subjects with dopaminergic dysfunction confirmed by SPECT are available. One patient with unilaterally reduced DaT activity (Paviour et al. 2006) did not respond to levodopa 500 mg t.i.d. and in another one with bilateral dopaminergic deficiency “poor response” was noted (Sung et al. 2009). However, in another patient who showed complete absence of DaTSCAN binding in striatum, 2.8 mg of pramipexole significantly ameliorated rest tremor and other tremor components improved when levodopa (600 mg/day) was added (Seidel et al. 2009). Only transient effect of combine dopaminergic therapy (lewodopa up to 900 and 3 mg of cabergoline daily) was observed in subject with posttraumatic HT and unilateral lack of DAT radiotracer binding revealed by SPECT (Reese et al. 2011).

The results of functional neuroimaging studies and the clinical data concerning effectiveness of dopaminergic therapy are inconsistent and doubts remain as to the accuracy of the hypothesis that że lesion of nigrostriatal dopaminergic system is a sine qua none condition for HT development. In recently published (Rydz et al. 2015) case of Holmes tremor related to anti-Yo cerebellar degeneration—uncommon pathology for HT—postmortem examination revealed apparently normal substantia nigra and striatum.

We present three patients with diagnosis of HT established according to the diagnostic criteria and with no involvement of pre- and postsynaptic part nigrostriatal system as shown by SPECT-DaTSCAN and SPECT-IBZM studies. The current clinical diagnostic criteria of HT are presumably insufficiently specific and when using them we identify patients both with and without the involvement of dopaminergic system. These two groups may represent tremor disorders of similar phenomenology but of different pathomechanism. We suggest that functional imaging studies of nigrostriatal dopaminergic system as well as response to levodopa and dopamine agonists should be an important part of evaluation process of subjects with clinical diagnosis of HT.
